# Unveiling the Mechanisms of Digital Technology in Driving Farmers’ Green Production Transformation: Evidence from China’s Watermelon and Muskmelon Sector

**DOI:** 10.3390/foods13233926

**Published:** 2024-12-05

**Authors:** Adiljan Musajan, Qingning Lin, Dawei Wei, Shiping Mao

**Affiliations:** Institute of Agricultural Economics and Development, Chinese Academy of Agricultural Sciences, Beijing 100081, China; 82101222510@caas.cn (A.M.); linqingning@caas.cn (Q.L.); 82101231410@caas.cn (D.W.)

**Keywords:** pesticides, fertilizers, sustainable agriculture

## Abstract

Leveraging the transformative potential of digital technologies to support the farmers’ green production transformation (FGPT) is a critical catalyst for facilitating the accelerated green transformation and upgrading of agricultural systems, thereby achieving high-quality agricultural development. Using survey data from major watermelon- and muskmelon-producing regions in Henan, Shandong, and Xinjiang, and employing Ordinary Least Squares (OLS), Propensity Score Matching (PSM), and the Logit model, this study examines the mechanisms through which digital technology influences FGPT. The findings reveal that the adoption of digital technology significantly reduces farmers’ use of pesticides and fertilizers, with a particularly pronounced effect on pesticide reduction. Heterogeneity analysis indicates that the impact of digital technology on reducing pesticide and fertilizer usage varies across regions: while its adoption leads to significant reductions in Henan and Shandong, the positive effects in Xinjiang remain statistically insignificant. Mechanism analysis shows that digital technology enhances FGPT by reducing information asymmetry, improving market access, and enhancing precision management practices. Based on these findings, this study recommends expanding awareness campaigns to accelerate the adoption of digital technology, enhancing digital infrastructure to bridge the urban–rural digital divide, and optimizing digital technology promotion systems. These actions can be taken alongside the implementation of economic incentives and compensation mechanisms. The insights and policy recommendations from this research provide valuable guidance for China and other countries seeking to leverage digital technology for sustainable agricultural development.

## 1. Introduction

The promotion of green development in agriculture represents a profound transformation in agricultural development strategies. Over the past 40 years, China’s agricultural growth has heavily relied on chemical fertilizers and pesticides, leading to ecological degradation and food safety concerns, making the transition to sustainable agricultural practices an urgent priority [1,2]. Farmers’ green production transformation (FGPT) serves as a critical approach to advancing sustainable agriculture by encouraging a shift from traditional to green production methods. FGPT emphasizes environmental sustainability throughout the production process—before, during, and after harvest—by adopting eco-friendly, resource-efficient, and circular approaches in production inputs, technologies, and management strategies. This transition supports scientific decision-making, demand–supply alignment, intelligent monitoring, and the expansion of sales channels, while focusing on resource conservation and environmental protection, ultimately enhancing agricultural quality, economic efficiency, and farm income [3,4,5,6,7,8]. FGPT provides an effective response to the unsustainable economic growth model linked to pollution and opens a new avenue for sustainable economic growth [9]. However, small-scale farmers often face limitations related to agricultural risks, market access, and production costs, which dampen their motivation to pursue green production. Identifying and promoting drivers of FGPT is therefore essential to the success of green agricultural development, holding significant importance for improving both industrial economic benefits and ecological outcomes.

As a high-value economic crop, watermelon and muskmelon (hereafter referred to as “melons”) hold a vital role in China’s agricultural economy, contributing significantly to agricultural restructuring, supporting farm incomes, and meeting growing market demand [10]. According to the Food and Agriculture Organization (FAO), China was the world’s largest producer of watermelons and muskmelons in 2022, accounting for 58.2% of global production. The country’s average yield per unit area was 1.3 times the global average, leading the world in productivity. However, recent years have seen China’s melon industry constrained by a “price ceiling” and a “cost floor”, along with pressures related to natural resource limitations and environmental protection demands. Together, these factors have gradually eroded both the economic and ecological benefits of melon production [11]. In terms of cost and profitability, melon farming is highly labor-intensive, with increasing prices for agricultural inputs such as plastic film, pesticides, and fertilizers, alongside rising labor costs, leading to higher production expenses. Additionally, issues such as concentrated market supply, inconsistent product quality, and difficulties in selling produce in primary production areas have led to a sharp drop in market prices, reducing overall profitability. In terms of supply and demand, the supply elasticity of melons is relatively low, meaning that their output is not very responsive to price changes. At the same time, melons have a relatively high demand elasticity, with many other fruits and vegetables serving as substitutes. As China transitions toward high-quality development, consumers increasingly seek premium, contaminant-free agricultural products. FGPT in melons not only reduces the use of chemical inputs and their environmental impact but also lowers input costs, improves product quality, and strengthens market recognition. This green transformation can expand sales channels, enhance brand recognition, and boost farmer income, enabling melon producers to increase profitability without reducing sales volume due to price increases. Therefore, FGPT in melons not only supports higher incomes and provides consumers with high-quality produce but also lays a solid foundation for the sustainable development of the melon industry. 

The factors influencing farmers’ green production transformation (FGPT) have become prominent topics in academic research and can generally be categorized as internal and external. Internal factors primarily relate to individual and household characteristics, including gender, age, education level, environmental awareness, and risk preference. For instance, gender and educational background significantly affect farmers’ understanding of green production practices [12,13]. Household income, labor structure, and farm size also directly impact farmers’ capacity for FGPT [14]. Time preference and risk tolerance play a critical role in FGPT decision-making. When the short-term benefits of green practices are not immediately evident, farmers often tend to stick to traditional production methods, resulting in limited motivation to adopt FGPT initiatives [15,16]. External factors encompass globalization government policies, technical training, agricultural subsidies, and the application of digital technologies. Globalization can significantly affect digital technology adoption in all countries [17]. Government policies [18,19], financial subsidies [7,20], and training in green practices [21] help to lower the barriers to green technology adoption, enhancing farmers’ understanding and adoption of sustainable agricultural methods. Moreover, digital technologies, as a hallmark of technological change in agriculture, drive FGPT and enhance overall green productivity through spillover effects that increase efficiency and sustainability [22,23,24]. Existing studies confirm that digital technologies accelerate agricultural innovation [25], improve technical efficiency [26], and raise human capital, thereby optimizing resource allocation. They also contribute to reducing energy consumption, lowering emissions, promoting mechanization, enhancing environmental performance, and increasing market transparency, all of which foster FGPT [27,28,29].

Amid the rapid development of the digital economy, digital technology has found increasingly widespread application in agriculture. Emerging technologies such as the Internet of Things, big data, and digital platforms are fundamentally reshaping agricultural production, distribution, and consumption, driving a shift from traditional to modernized agricultural practices [30,31,32,33,34]. From a global perspective, German agriculture took a leading role in the adaptation of digital technologies years ago and has maintained this role to date, effectively promoting agricultural digitalization [35]. However, significant challenges that need to be addressed in the digitization of African agriculture include scarce resources, limited expertise and training, a lack of digital infrastructure, data privacy and security issues, and farmer resistance [36]. Digital technology adoption plays an important role in China. Digital technology is not only a major driver of the digital economy but has also become a core factor in advancing high-quality agricultural development [37,38]. In recent years, the swift growth of e-commerce and smart agriculture technologies has diversified sales channels for watermelon and muskmelon, connecting major production areas in Shandong, Henan, and Xinjiang to markets in major cities like Beijing, Shanghai, Guangdong, and Shenzhen. This expansion has helped to alleviate the issue of unsold produce in primary production regions, facilitating income growth for growers and highlighting digital technology’s vast potential to integrate and streamline the agricultural value chain. Accordingly, the application of digital technology among watermelon and muskmelon farmers is becoming a crucial pathway for promoting sustainable development in the sector [39]. However, despite the expanding use of digital technology in agriculture, challenges such as the digital divide and farmers’ capacity to adopt new technologies continue to hinder agricultural digitalization [40,41,42]. As highly market-sensitive producers, watermelon and muskmelon farmers’ experiences with digital technology in terms of facilitating FGPT can provide valuable insights for advancing green agricultural development. Therefore, exploring the mechanisms by which digital technology’s application among watermelon and muskmelon farmers drives FGPT has significant theoretical and practical value. 

This study investigates the impact of digital technology on farmers’ green production transformation (FGPT) within the context of the watermelon and muskmelon industry in China, focusing on 552 farmers in Henan, Shandong, and Xinjiang. It further provides a systematic analysis of the underlying mechanisms. The selected research regions are representative in terms of both industry scale and regional characteristics. Together, these three provinces produce 31 million tons of watermelon and muskmelon annually, accounting for 36.5% out of the national total. While Shandong and Henan are characterized by intensive management practices, Xinjiang leverages its favorable natural conditions for large-scale open-field cultivation. These regional differences offer a robust foundation for analyzing the heterogeneity in FGPT outcomes across varying contexts. Additionally, compared to other agricultural sectors, the watermelon and muskmelon industry is highly market-driven, making it an ideal entry point for examining how digital technology facilitates FGPT. This study specifically incorporates the most common and effective digital technologies employed by farmers to address agricultural production and marketing challenges. By scientifically selecting key mechanism variables, such as market access, information accessibility, and precision management, this study not only uncovers the pathways through which digital technology influences FGPT but also enriches the existing body of literature on this topic. The findings of this study provide valuable insights for China and other countries, particularly developing nations, in terms of effectively utilizing digital technologies to promote sustainable agricultural development and drive the transformation of agriculture in a more green and sustainable direction. These results also offer a feasible pathway and practical case studies for global agricultural green transformation, with significant implications for future policy and practice.

The remainder of this paper is structured as follows: Section 2 outlines the theoretical framework and mechanism analysis employed for digital technology and FGPT; Section 3 details the data and empirical strategies utilized; Section 4 presents the empirical results; and Section 5 and Section 6 discuss the findings and offer conclusions. 

## 2. Theoretical Analysis and Research Hypothesis

### 2.1. Theoretical Analysis of Digital Technology Application and Farmer’s Green Production Transformation (FGPT)

The application of digital technology is reshaping agricultural production methods, providing strong momentum for FGPT [43]. Watermelon and muskmelon production, as labor-intensive and resource-demanding tasks, faces rising production costs year by year, making resource conservation and cost control increasingly important. From a theoretical perspective, the application of digital technology can enhance resource utilization efficiency, improve production process transparency, and facilitate a transition from experience-based to data-driven management for melon farmers [44].

Firstly, digital technology promotes green production practices by enhancing productivity and resource efficiency [45]. Precision agriculture technologies, such as intelligent monitoring systems, drones, and IoT devices, allow farmers to fertilize and irrigate according to actual field needs, reducing unnecessary resource input [46]. This data-driven management approach effectively reduces the use of fertilizers and pesticides, minimizes environmental pollution risks, and decreases agricultural production’s reliance on natural resources. 

Secondly, digital technology enhances transparency and controllability throughout the production process [47]. Real-time monitoring and data analysis allow farmers to better understand the input–output conditions at each stage of production. This transparent management framework enables farmers to develop more scientific planting plans.

Data-based decision-making not only reduces uncertainty but also curbs overexploitation and blind investment, providing strong support for green production practices.

Lastly, digital technology enables a shift from experience-based to data-driven agricultural management [48]. Traditional agricultural decisions often rely on farmers’ personal experience, whereas digital technology quantifies critical variables, enabling farmers to make more rational judgments based on data. This transition improves the scientific rigor and sustainability of agricultural practices, curbing resource waste and laying a solid foundation for FGPT.

Further, this study treats digital technology as a new production factor and provides mathematical evidence of its role in promoting FGPT through an expanded production function model. Let us assume that a farmer’s production functions as follows:

(1) Q=f(X,D)

where *Q* represents output, *X* represents traditional inputs (e.g., labor, fertilizers), and *D* denotes digital technology. The function *f* adheres to the law of diminishing marginal returns:

(2) $$\frac{\partial f}{\partial X}>0,\;\frac{\partial^{2}f}{\partial X^{2}}<0,\;\frac{\partial f}{\partial D}>0,\;\frac{\partial^{2}f}{\partial D^{2}}<0$$

The introduction of the digital technology *D* optimizes resource allocation and reduces reliance on traditional inputs *X*, indicating a substitution effect. As digital technology use increases, farmers rely less on traditional inputs:

(3) $$\frac{\partial^{2}f}{\partial X\partial D}<0$$

If green production aims to maximize output *Q* and environmental friendliness *G*, environmental friendliness *G* depends on the extent to which chemical and resource inputs are reduced and is assumed to be negatively correlated with the use of traditional inputs. Environmental friendliness G is expressed as follows:

(4) $$G=g(X),\;\;\;\;g^{\prime }(X)<0$$

A farmer’s combined utility function is therefore expressed as follows:

(5) U=f(X,D)+ g(X)

Farmers maximize this utility by selecting optimal levels of *X* and *D*:

(6) $$\underset{X,D}{\max}\;\;U=f(X,D)+\;g(X)$$

The first-order conditions for X and D can be determined as follows:

(7) $$\frac{\partial U}{\partial X}=\frac{\partial f}{\partial X}+g^{\prime }(X)=0,\;\;\frac{\partial U}{\partial D}=\frac{\partial f}{\partial D}=0$$

These conditions illustrate that farmers’ choices of *X* and *D* balance marginal production gains with environmental benefits. Digital technology adoption optimizes resource allocation, reduces the overuse of traditional inputs, and enhances environmental friendliness *G*. Thus, the application of digital technology can drive FGPT by boosting efficiency and reducing resource consumption.

Based on the above analysis, we propose the following hypothesis:

**H1.**  *The application of digital technology can promote FGPT among watermelon and muskmelon farmers.*

### 2.2. Mechanisms of Digital Technology in Promoting FGPT

The application of digital technology plays a pivotal role in advancing farmers’ green production transformation (FGPT) within the watermelon and muskmelon industry [49]. Drawing on information economics, market access theory, and production factor optimization theory, this study builds a theoretical framework that examines the mechanisms by which digital technology drives FGPT. Through enhancing information equity and market access capabilities, and by implementing precision management, digital technology generates multiplier effects, knowledge spillovers, and price premiums, enabling watermelon and muskmelon farmers to make scientific decisions, match input supply and demand, optimize in-process monitoring, and expand sales channels throughout the production cycle. This integration reduces resource waste and environmental impacts, fostering a comprehensive transition to green production practices [50,51,52]. 

First, by enhancing information accessibility, digital technology addresses information asymmetry, a significant barrier to FGPT [49]. According to information economics, asymmetrical information can inhibit farmers from making sound production decisions. In traditional farming, watermelon and muskmelon growers often lack access to real-time data on field conditions and pest risks, resulting in excessive pesticide and fertilizer use, which leads to resource waste and environmental pollution. Through the Internet of Things, big data, and intelligent platforms, digital technology provides farmers with essential information on weather changes, soil nutrients, and pest control, reducing information asymmetries significantly. Additionally, digital technology enables farmers to track market supply–demand relationships, price fluctuations, and customer feedback in real time, improving their responsiveness to market needs. By analyzing sales data on digital platforms, farmers can adjust planting plans promptly, minimizing the risks of overproduction or unsold inventory. Moreover, customer feedback given on online platforms allows farmers to understand consumer preferences regarding quality and taste, enabling ongoing product improvements and fostering a strong brand reputation.

Digital technology also enhances market access for watermelon and muskmelon farmers, accelerating FGPT [43]. From the perspective of market access theory, access to larger markets not only increases economic returns but also motivates farmers to adopt more sustainable, eco-friendly practices. Specifically, digital platforms reduce the distance between growers and end consumers, allowing farmers to bypass traditional intermediaries and directly sell their green products. This direct sales model reduces intermediary costs, increases farmer income, and strengthens farmers’ control over pricing and bargaining power [50]. Furthermore, by showcasing and promoting green products on e-commerce platforms, farmers can benefit from price premiums associated with the health and sustainability features of green products, providing an economic incentive for FGPT. This closed-loop system of “green production–online sales–profit increase” offers robust market support for green transformation. 

Finally, digital technology advances FGPT by improving precision management [51]. Designed based on production factor optimization theory, the scientific and efficient allocation of resources—such as land, water, labor, and capital—leads to knowledge spillover effects. Smart agricultural technologies, such as drone monitoring, precision irrigation, and intelligent greenhouses, optimize the production process through automated management, ensuring crops receive inputs as needed and that resources are utilized efficiently. This approach reduces chemical inputs, lowers environmental impacts, and enhances production efficiency. Digital technology thus shifts watermelon and muskmelon farmers from traditional experience-based management to a scientific, data-driven model, enabling informed decision-making and aligning input supply with demand, thereby strongly supporting the greening of agricultural production. 

**Figure 1:**
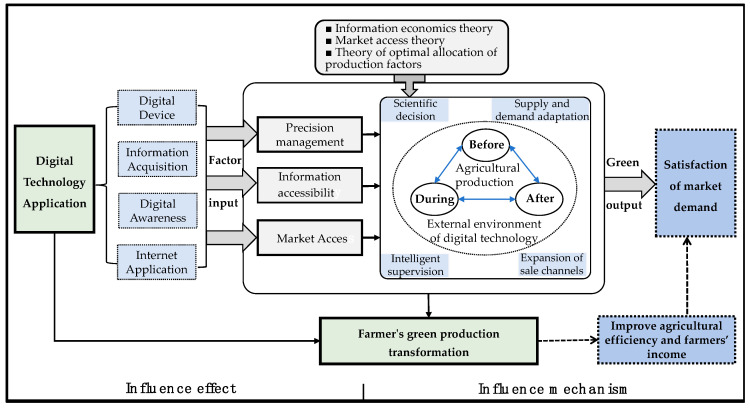
Theoretical analysis framework.

Based on the above theoretical analysis, we construct the theoretical framework depicted in Figure 1 and proposes the following hypothesis:

**H2.**  *Digital technology promotes FGPT among watermelon and muskmelon farmers by enhancing information accessibility, expanding market access, and facilitating precision management.*

## 3. Materials and Methods

### 3.1. Data Description

#### 3.1.1. Study Area

This study selected Henan, Shandong, and Xinjiang as survey regions. These are significant watermelon and muskmelon production areas in China, representing key characteristics and scales within the industry. In 2022, these three regions collectively produced 30.96 million tons of melons, accounting for 36.5% out of national production and ranking first, second, and fourth in output, respectively. Henan and Shandong primarily use intensive management practices, while Xinjiang, leveraging its favorable natural conditions, focuses on large-scale open-field cultivation. This regional variation provides a solid foundation for analyzing the heterogeneity in FGPT among melon farmers. 

Xinjiang, located in the western part of China, has an arid climate with abundant sunlight, significant day–night temperature fluctuations, and fertile sandy soil, making it ideal for high-quality melon production. Well-known melon varieties such as Hami melon, Weili melon, and Kashgar melon have provided strong economic impetuses to the region’s development. 

Henan and Shandong, located in eastern China, have mild climates, ample sunlight, fertile soil, good drainage, and well-developed agricultural infrastructure, creating favorable conditions for the growth of melons and watermelons. Henan’s Malianzhuang melon, recognized as a geographical indication product, has become a highlight and recognizable variety of local agricultural product due to its superior geographic environment, rich cultivation experience, and high-quality produce. In 2021, the geographical indication product value of Changle watermelon in Shandong Province reached CNY 4.317, ranking first in China in terms of watermelon brand value. The outstanding performance of these regions in the melon industry provides rich samples and a strong observational basis for this study.

#### 3.1.2. Data Source

The survey was conducted in July 2023, covering six major watermelon and muskmelon growing areas across Shandong, Henan, and Xinjiang. Regional representativeness and heterogeneity were considered when selecting these areas. Xinjiang, located in western China, and Henan and Shandong, located in eastern China, are about 3500 km apart. This regional difference provides a good foundation for analyzing the heterogeneity in the green production transformation of melon farmers. 

In terms of sample selection, a combination of stratified and random sampling was used to select 552 samples. A total of 28 administrative villages were randomly chosen, with 20 to 30 melon farmers selected from each village for in-depth interviews. This sampling approach ensured that the surveyed sample was representative of the melon industry in these regions. The survey questionnaire covered basic household characteristics, production models, and the use of digital technologies, providing solid data support for exploring the relationship between the adoption of digital technology and farmers’ green production transformation.

### 3.2. Variable Definition and Descriptive Statistics

#### 3.2.1. Core Explanatory Variable: Digital Technology Application (Digital)

The concept of digital technology application is broad, necessitating tailored measures based on specific contexts. In small-scale farming, commonly used digital technologies include internet access, mobile applications, and digital platforms, which are essential for managing production and sales challenges [53]. Drawing on existing research, this study defines digital technology application among melon farmers as a composite measure comprising four dimensions: device ownership, information acquisition, digital platform awareness, and practical application [54,55,56]. Specifically, digital technology application is measured using four binary variables (0–1), which are defined as follows:

1. Digital device ownership—whether farmers own digital devices such as smartphones, tablets, or computers.
2. Internet information acquisition—whether farmers access melon cultivation or production information through the internet.
3. E-commerce and digital platform awareness—whether farmers are familiar with the functions and usage of e-commerce platforms like Taobao, Pinduoduo, and Douyin.
4. Internet sales application—whether farmers use online platforms to sell melons.

#### 3.2.2. Dependent Variable: Farmers’ Green Production Transformation (FGPT)

Reducing the use of chemical inputs is a critical pathway in terms of advancing farmers’ green production transformation (FGPT). To measure FGPT, this study uses the most direct input indicators: the per mu usage of pesticides and fertilizers [25]. Reductions in chemical inputs serve as key metrics of agricultural sustainability, capturing farmers’ efforts to lessen environmental impacts and promoting sustainable practices [57,58]. By precisely measuring the per mu usage of fertilizers and pesticides, this research provides a measurable assessment of the extent to which farmers are engaging in the green production transition.

#### 3.2.3. Mechanism Variables

To examine how the application digital technology application drives FGPT, this study identifies three key mechanism variables based on theoretical analysis and existing research: information accessibility, market access, and precision management. The definitions and measurement methods for each mechanism variable are as follows:

1. Information accessibility (Inform): this variable reflects the frequency with which farmers access technical and operational information, particularly through the internet [50].
2. Market access (Market): This variable captures farmers’ ability to expand sales channels and reach broader markets through digital technology [57]. A higher share of e-commerce income indicates proactive market engagement, which helps farmers to enhance their green product’s market premium and encourages a shift toward FGPT.
3. Precision management (Precise): This variable assesses whether farmers use technology for efficient resource allocation and the precise management of inputs [59,60]. These technologies help to reduce resource waste and chemical inputs in production, increasing environmental sustainability and facilitating FGPT.

#### 3.2.4. Control Variables

Following prior research and considering the specific needs of this study, individual characteristics, household characteristics, and production characteristics are selected as the control variables [61,62].

1. Individual characteristics: These include gender (Gender), age (Age), and education level (Edu). Gender differences may influence roles in agricultural production, potentially leading to variations in technology adoption and green production behaviors. Age may affect reliance on traditional methods, with younger farmers more likely to adopt digital technologies. Education level is also included, as higher education levels are associated with greater openness to new production technologies.
2. Household characteristics: These variables encompass household labor size (Labor), non-agricultural income (Income), and village leader status (Identity). The household labor size determines the practical capacity for production and technology adoption. Non-agricultural income reflects the degree of economic diversification in a household; higher non-agricultural income may increase risk tolerance and the willingness to innovate. Village leader status could enhance sensitivity to policy and technology, influencing decision-making behaviors.
3. Production characteristics: These include planting experience (Plant) and planting scale (Scale). Planting experience captures the farmer’s level of expertise, as experienced farmers may be more inclined to explore green technologies. Planting scale, measured by the area of watermelon and muskmelon cultivation (mu), is used to control for differences in technology adoption and green production across farmers of varying scales. Descriptive statistics for all variables are provided in Table 1.

### 3.3. Research Methods

#### 3.3.1. Baseline Regression Model

Since FGPT is represented by per-unit-area fertilizer and pesticide usage, which are continuous variables, a multiple linear regression model, estimated using Ordinary Least Squares (OLS), is appropriate for the analysis. The baseline model is specified as follows:

(8) $$FGPT_{i}=a_{0}+cDigital_{i}+a_{1}Control_{i}+\varepsilon_{i}$$

where *i* denotes individual melon farmers, FGPT represents the degree of farmers’ green production transformation, *Digital_i_* is the core explanatory variable of digital technology application, control includes a series of control variables related to personal, household and production characteristics, and *ε* represents the random error term, encompassing all stochastic factors that cannot be explained by the control variables and the application of digital technologies. The intercept term α_0_ represents the baseline level of green production transformation when all explanatory variables are set to zero. The coefficient *c* represents the effect of the application of digital technologies on the degree of green production transformation. If the estimated value of *c* is statistically significant and *c* = −0.5, this indicates that, for each unit increase in the application of digital technologies, the degree of green production transformation (such as the reduction in fertilizer and pesticide use) decreases by 0.5 units, thereby contributing to the green transformation process.

#### 3.3.2. Propensity Score Matching (PSM)

Digital technology applications are not randomly distributed among farmers but rather reflect farmers’ self-selection based on individual conditions, resources, and environmental factors. Such self-selection can lead to systematic differences within the sample, resulting in endogeneity issues. Without addressing this, estimates from traditional regression models can be biased due to selection bias. To control for these biases, this study employs Propensity Score Matching (PSM), a method that balances treatment and control groups, making them comparable by accounting for non-random differences based on farmer characteristics.

PSM offers several advantages: first, it balances differences between treated (digital technology adopters) and untreated farmers in terms of control variables, enabling more accurate comparisons; second, it focuses specifically on the causal effect of digital technology application, reducing biases originating from endogeneity; third, PSM does not rely on specific functional forms, enhancing the robustness of results. 

This study begins by using a Logit model to predict each farmer’s propensity score in terms of adopting digital technology:

(9) $$P(D_{i}=1\left|X_{i}\right.)=\frac{e^{X_{i}\delta }}{1+e^{X_{i}\delta }}$$

where *P*(*D_i_ =* 1*∣X_i_*) is the probability that farmer *i* adopts digital technology when accounting for the control variable *X_i_*. The propensity score is then used to match treated and control groups through nearest-neighbor, radius, or Mahalanobis distance matching to ensure the balance of control variables between the treatment and control groups.

The average treatment effect on the treated (ATT) is then calculated using the matched data as follows:

(10) ATT=(1/Nt)×∑(Yi1−Yi0)

where *N_t_* is the sample size of the treatment group, *Y_i1_* represents the outcome variable for treated individuals, and *Y_i_*_0_ is the outcome for comparable untreated individuals. 

#### 3.3.3. Mechanism Testing Model

To test the mechanisms through which digital technology influences FGPT, we extend the baseline regression model to include mechanism variables, resulting in the following model:

(11) Mi=α2+αDigitali+α3Controli+εi

where *M_i_* represents mechanism variables such as information accessibility, market access, and precision management. A significant estimated coefficient *α* would indicate the validity of that specific mechanism. If the estimated value of α is statistically significant and positive, it indicates that the application of digital technologies has a positive effect on the mechanism variables (such as information accessibility, market access, etc.).

Considering that mechanism variables like information accessibility, market access, and precision management are discrete variables, we also apply a Logit regression model to test robustness. The general form of the Logit model is as follows:

(12) $$P(M_{i}=\left.1\right|X_{i})=\frac{1}{1+e^{-(\gamma_{0}+\gamma_{1}Digital_{i}+\gamma_{2}X_{i})}}$$

where *P(M_i_ =* 1*∣X_i_)* denotes the probability of a farmer adopting a particular mechanism (such as improved information accessibility or market access) given the control variables *X_i_*. $\gamma_{0}$ represents the intercept term; $\gamma_{1}$ denotes the coefficient measuring the impact of digital technology (*Digital_i_*) adoption on the mechanism variables; $\gamma_{2}$ corresponds to the regression coefficients of the control variables *Xi*; and *e* signifies the natural constant. This Logit approach is suitable for binary or discrete dependent variables and ensures the robustness of the mechanism analysis. In the Logit model, the coefficient is interpreted as the log odds of a change in probability. For example, if the estimated value of $\gamma_{1}$ is positive, it indicates that the application of digital technologies increases the probability of farmers adopting a particular mechanism (such as information accessibility). 

## 4. Results

### 4.1. Baseline Regression

Table 2 presents the baseline regression results from the examination of the impact of digital technology adoption on the per mu use of pesticides and fertilizers among farmers. Models (1) and (4) provide the results of regressions with the core explanatory variable—digital technology adoption—in relation to pesticide and fertilizer use, respectively. Models (2) and (5) incorporate control variables, while Models (3) and (6) apply a logarithmic transformation to pesticide and fertilizer use to test the robustness of the results.

The findings reveal that digital technology adoption significantly reduces per mu pesticide and fertilizer usage, regardless of whether control variables are included or a logarithmic transformation is applied. These results provide preliminary support for Hypothesis H1, underscoring the critical role of digital technology in facilitating FGPT at the micro level.

Among the control variables, age shows a significant positive association with pesticide and fertilizer usage, indicating that older farmers are more reliant on these inputs, which hinders FGPT. Conversely, planting scale demonstrates a significant negative relationship, suggesting that larger planting areas help to reduce pesticide and fertilizer use, thereby promoting FGPT. A higher non-agricultural income within a household is associated with increased pesticide and fertilizer use, which may reflect a reduced focus on optimizing agricultural input efficiency. In contrast, households with members serving as village officials and those with higher levels of education are more likely to reduce pesticide and fertilizer usage, facilitating FGPT.

### 4.2. Addressing Endogeneity

Since the decision to adopt digital technology is not random but rather a self-selected process influenced by observable characteristics such as farmers’ age, gender, and education level, as well as potentially unobservable factors like personal traits, there may be endogeneity issues in the regression results due to sample self-selection, leading to potential biases in the finding. To address this, we apply Propensity Score Matching (PSM) to ensure robustness and account for endogeneity, reducing bias in the estimated effects. 

We first use a Logit model to estimate the probability of digital technology adoption, incorporating control variables as covariates. PSM is then applied to estimate the Average Treatment Effect on the Treated (ATT) with regard to farmers adopting digital technology. As shown in Table 3, across various matching methods—kernel, nearest-neighbor, radius, and Mahalanobis distance matching—the ATT results indicate that digital technology adoption continues to significantly reduce the use of pesticides and fertilizers per unit area, with all effects significant at the 1% level. These findings align with the baseline regression results, confirming the robustness of the conclusions. 

### 4.3. Regional Heterogeneity Analysis

The previous analysis confirmed the impact of digital technology adoption on farmers’ pesticide and fertilizer usage per unit area. However, given China’s vast regional diversity, variations in natural conditions, production practices, technological awareness, and agricultural extension services likely influence the effect of digital technology across regions. To capture these regional differences, we conducted a heterogeneity analysis based on three subsamples from the surveyed regions, with results shown in Table 4. The findings indicate that digital technology adoption significantly reduced pesticide and fertilizer usage among farmers in Henan and Shandong. However, this effect was not observed among melon farmers in Xinjiang.

### 4.4. Mechanism Analysis

Based on prior theoretical analysis and hypotheses, we explore how digital technology influences FGPT through three pathways: information accessibility, market access, and precision management. Given the discrete nature of the mechanism variables, we used both OLS and Logit models to test robustness. The results are presented in Table 5.

1. Information accessibility: In the information accessibility pathway, the OLS model shows a coefficient of 0.316 for digital technology adoption, which is significant at the 10% level, indicating that technology adoption significantly increases farmers’ access to agricultural and business information. The Logit model further corroborates this conclusion, with a coefficient of 0.744 for digital technology, which is significant at the 1% level. These results suggest that digital technology effectively broadens farmers’ access to essential information, enhancing their capacity to obtain critical production data.
2. Market access: In the market access pathway, the OLS model coefficient for digital technology adoption is 0.130, which is significant at the 5% level, indicating that technology adoption significantly increases farmers’ likelihood of using e-commerce platforms for sales. The Logit model also shows a positive coefficient of 0.877, which is significant at the 5% level, reinforcing the idea that digital technology helps farmers to access broader markets and streamline sales channels, thereby supporting the sale of green products.
3. Precision management: For precision management, the OLS model shows a significant coefficient of 0.422 for digital technology at the 1% level, indicating that digital adoption significantly encourages farmers to use precision management practices in production. In the Logit model, the coefficient is 0.683 and this is also significant at the 1% level. This positive coefficient reflects the nonlinear nature of precision management’s significance in the Logit model, suggesting that digital tools and data enable more efficient resource management, reducing waste and environmental impacts, and thereby facilitating FGPT.

In summary, these analyses confirm that digital technology adoption influences FGPT through information accessibility, market access, and precision management. Digital technology not only provides the tools for information acquisition and market entry but also enables efficient resource use and precise input management. The consistency between the OLS and Logit models further verifies the robustness of these transmission mechanisms, providing strong support for the core conclusions of this study.

## 5. Discussion

In order to explore the impact and mechanisms of digital technology adoption on the green production transformation of watermelon and Muskmelon growers, this study utilizes empirical data from field research within China’s watermelon and melon industry to conduct both theoretical analysis and empirical testing. Compared to previous studies, this paper makes marginal contributions in the following three aspects.

First, while previous studies have largely used macro-level data to demonstrate the influence of digital technology on green transformation [24,25,26,27], this study comprehensively considers all aspects of agricultural production, from agricultural factor inputs to green outputs, and constructs a theoretical analysis framework on how digital technology promotes GTFP based on existing research and the definition of GTFP. Additionally, the study advances the analysis by using micro-level survey data. By examining farmers’ production behavior and market responses, we provide a detailed view of how digital technology drives FGPT. Our findings indicate that digital technology adoption significantly reduces pesticide and fertilizers usage, reducing pesticide use in particular. This supports previous research on digital technology’s role in improving productivity and optimizing resource utilization [26,30,59]. However, unlike prior studies that focused solely on technical efficiency, this study not only verifies the direct effects of digital technology on FGPT among melon farmers but also identifies the mechanisms—such as market access, information accessibility, and precision management—through which it influences green production, adding depth to the understanding of digital technology’s role in FGPT.

Second, considering regional differences in natural conditions, production practices, technological awareness, and agricultural extension services, this study examined the heterogeneous effects of digital technology on FGPT across different regional backgrounds. The results indicate that digital technology adoption significantly reduced pesticide and fertilizer use among melon farmers in Henan and Shandong, whereas no significant effects were observed in Xinjiang. This may be attributed to Xinjiang’s relatively lower level of economic development and limited digital infrastructure, and the lower levels of digital literacy prevailing among farmers. Additionally, Xinjiang’s arid climate and reduced pest prevalence lessen the reliance on pesticides, constraining the impact of digital technology on FGPT. These findings corroborate the notion that external factors significantly influence the effectiveness of digital technology adoption [16,17,18].

Third, by focusing on the highly market-oriented melon industry, this study provides a micro-level analysis of how digital technology facilitates FGPT through market premium mechanisms. This finding extends Griliches’ (1957) theory on the economic motivation for technology adoption, offering empirical support for a market behavior perspective [27]. Additionally, this study confirms that farmer characteristics, such as gender and education level, impact FGPT [12,13]. Findings show that digital technology improves information flow and market access, which in turn enhances farmers’ motivation for green production. This suggests that, in addition to individual traits, market conditions and technological innovation are also crucial drivers of FGPT.

It is important to acknowledge certain limitations in this study, despite its valuable findings on the relationship between digital technology applications and FGPT among watermelon and muskmelon growers. First, the research sample is confined to three major watermelon-producing regions in China and does not account for varying external factors such as digital infrastructure, natural environments, and relevant policies. Future research could encompass a broader range of regions and additional factors, further validating the intensity of the impact of digital technology adoption in advancing the FGPT. At the same time, a more in-depth analysis of the Xinjiang case could be used to supplement the discussion. Second, the data and methods used in this study have certain limitations. Regarding empirical methods, while propensity score matching (PSM) helps to control selection bias, it is limited by relying on observable variables and the assumption of common support, which may not fully account for unobserved confounding factors. As this study employs cross-sectional data, it does not capture the long-term effects of digital technology application on FGPT. There may also be errors in data collection, such as misinterpretations of the questionnaire by farmers or biases in their perceptions. Future studies could use longitudinal data to explore the dynamic impacts of digital technology on the evolving behavior of watermelon and muskmelon growers in their green production transition. Third, the dependent variable “digital technology” in this study is a composite indicator that covers digital technologies used across various stages of production and consumption. However, the study does not distinguish between the specific types of digital technologies, which is a limitation of this work. Future research could explore the differential impacts of specific digital technologies at different stages of the agricultural process on the green production transformation of farmers.

## 6. Conclusions

This study constructs a theoretical mechanism framework using information economics, market access theory, and production factor optimization theory to analyze how digital technology influences FGPT. Drawing on data collected from primary watermelon-producing regions in Henan, Shandong, and the Xinjiang Uygur Autonomous Region, we employed baseline regression and Probit models to empirically validate our theoretical predictions, conducting comprehensive endogeneity and robustness checks. Additionally, this study considers China’s vast geographic variation, which includes diverse natural conditions, production modes, technological awareness, and agricultural extension practices, allowing for an exploration of regional heterogeneity.

This study reaches three key conclusions through baseline regression, regional heterogeneity analysis, and mechanism testing: (1) Digital technology application facilitates FGPT among watermelon and muskmelon growers, and this conclusion remains robust during endogeneity and robustness checks. (2) Regional heterogeneity analysis reveals that the effect of digital technology on FGPT differs significantly across regions, with a notable reduction in pesticide and fertilizer use in Henan and Shandong, while in Xinjiang the impact of digital technology on pesticide and fertilizer usage remains insignificant. (3) Mechanism analysis shows that digital technology promotes FGPT via three pathways—enhancing information accessibility, market access, and precision management. Digital technology empowers inclusive access to information, improving the growers’ ability to obtain production techniques and market insights, effectively reducing information asymmetry. It also expands growers’ access to e-commerce platforms, facilitating entry into broader markets. Furthermore, digital tools enhance the efficiency of resource allocation and input management through precise irrigation and pest control, elevating precision management capabilities and ultimately advancing FGPT. The conclusions of this study provide valuable insights for digitalization in other sectors. Additionally, the findings offer practical recommendations for other countries, especially developing nations, to allow them to promote agricultural digitalization.

This study offers the following policy recommendations:

1. It is necessary to broaden communication channels and accelerate digital technology adoption. Strengthening the dissemination of digital technology in rural areas can boost adoption rates among growers, enhancing their technical capacities. By emphasizing the economic and ecological benefits of digital technology, these initiatives can increase growers’ motivation to pursue green production.
2. It is necessary to accelerate digital infrastructure development and bridge the urban–rural digital divide. The impact of digital technology on FGPT varies significantly across regions. Expanding investments in rural digital infrastructure, enhancing network coverage, and upgrading digital facilities can ensure that growers in less developed areas benefit equally from digital tools. By narrowing the urban–rural divide regarding access to digital resources, rural development can progress in tandem with development in urban areas.
3. It is necessary to optimize extension systems and enhance economic incentives and compensation mechanisms. Establishing a centralized digital platform that integrates agricultural production, management, and market information, coupled with a green agricultural e-commerce platform, can amplify the multiplier effects of digital factors. This platform should provide quality certification and product traceability services. Throughout the production cycle, growers need access to timely and reliable information to support green production decisions and optimize resource allocation. Expanding market access with enhanced price premiums for sustainable products strengthens consumer trust in green practices. Additionally, given the economic costs and potential transition risks associated with adopting digital technologies, policies should address growers’ cognitive capacities and varying needs by offering risk-sharing and economic compensation programs. This approach helps to alleviate financial pressures, ensuring growers can achieve satisfactory expected returns from green production practices.

## Figures and Tables

**Table 1:** Descriptive statistics of variables.

Category	Symbol	Definition	Assignment	Mean	Std.dev.
Dependent variable	Fertilizer	The per-unit-area (per mu (1 mu = 666.67 m^2^)) use of fertilizers	Continuous variables	149.994	234.687
	Pesticides	The per-unit-area (per mu) use of pesticides	Continuous variables	7.976	26.418
	lnFertilizer	Logarithm of fertilizer weight	Continuous variables	4.569	0.869
	lnPesticides	Logarithm of pesticides weight	Continuous variables	0.724	1.798
Core explanatory variable	Digital	This study defines digital technology application among melon farmers as a composite measure comprising four dimensions: device ownership, information acquisition, digital platform awareness, and practical application.	Each of these binary variables is assigned a value of 0 or 1, and their sum forms a composite index (0–4)	2.955	0.371
Mechanism variables	Inform	The frequency with which farmers access technical and operational information, particularly through the internet.	1 = very often; 2 = often; 3 = sometimes; 4 = rarely; 5 = almost never	3.512	1.464
	Market	Farmers’ ability to expand sales channels and reach broader markets through digital technology.	1 = less than 30%; 2 = 30–60%; 3 = above 60%	1.483	0.550
	Precise	Farmers use technology for efficient resource allocation and precise input management.	1 = yes; 0 = no	0.768	0.422
Individual characteristics	Gender	Male or female	1 = male; 2 = female	0.945	0.226
	Age	Farmers’ age	Continuous variables	49.811	10.440
	Edu	Elementary, middle school, high school, college or above	1 = primary school; 2 = middle school; 3 = high school; 4 = university, and 5 = above	3.027	0.429
Household characteristics	Labor	The number of available laborers influences a household’s capacity for production and technology adoption.	Continuous variables	2.338	0.830
	Income	Total non-agricultural income	Continuous variables	4267.056	2874.041
	Identity	Yes or no	1 = yes; 0 = no	0.364	0.276
Production characteristics	Plant	The number of years a farmer has cultivated melons.	Continuous variables	8.294	16.573
	Scale	The area (in mu) under melon cultivation.	Continuous variables	7.497	19.460

**Table 2:** Baseline regression.

	(1)	(2)	(3)	(4)	(5)	(6)
Variables	Pesticides	Pesticides	lnPesticides	Fertilizer	Fertilizer	lnFertilizer
Digital	−61.13 ***	−53.48 ***	−1.863 ***	−137.9 ***	−99.65 ***	−0.294 ***
	(1.556)	(1.599)	(0.189)	(26.32)	(26.73)	(0.0980)
Gender		−3.257	0.0398		−69.04	−0.362 **
		(2.524)	(0.342)		(42.20)	(0.154)
Age		0.102 *	0.0166 **		4.332 ***	0.0191 ***
		(0.0575)	(0.0071)		(0.962)	(0.0036)
Edu		−0.318 **	−0.142 **		−0.114 *	−0.027 **
		(0.047)	(0.046)		(0.046)	(0.0131)
Labor		0.777	0.0219		−9.881	−0.0269
		(0.703)	(0.0852)		(11.76)	(0.0429)
Income		1.024	0.184 ***		1.077 **	0.144 **
		(0.505)	(0.386)		(0.393)	(0.0711)
Identity		0.0235	0.0170 ***		0.768	0.0038 **
		(0.0295)	(0.0035)		(0.493)	(0.0018)
Plant		−1.385	−0.106		−0.661	−0.0101
		(0.380)	(0.327)		(0.3932)	(0.0221)
Scale		0.121 ***	−0.0096 **		−1.098 *	−0.0094 ***
		(0.0350)	(0.0041)		(0.585)	(0.0035)
Constant	188.6 ***	184.0 ***	5.526 ***	557.5 ***	331.9 ***	4.988 ***
	(4.634)	(6.598)	(0.799)	(78.38)	(110.3)	(0.405)
Observations	552	552	552	552	552	552
R-squared	0.256	0.356	0.746	0.348	0.400	0.733

Note: * *p* < 0.1, ** *p* < 0.05, and *** *p* < 0.01. Robust standard errors are given in parentheses.

**Table 3:** Propensity score matching.

	Pesticides			Fertilizer		
Matching Method	Treatment Group	Control Group	ATT	Treatment Group	Control Group	ATT
Kernel matching	11.785	21.745	−9.960 ***	80.645	130.745	−50.100 ***
Nearest-neighbor matching	11.785	27.551	−15.766 ***	80.645	147.551	−66.906 ***
Radius matching	11.785	23.452	−11.667 ***	80.645	135.452	−54.807 ***
Mahalanobis matching	11.785	23.532	−11.747 ***	80.645	144.532	−63.887 ***

Note: *** *p* < 0.01. Robust standard errors are given in parentheses.

**Table 4:** Regional heterogeneity.

Variables	Henan	Shandong	Xinjiang	Henan	Shandong	Xinjiang
	lnPesticides			lnFertilizer		
Digital	−0.480 ***	−1.924 ***	−0.297	−0.234 ***	−0.220 ***	−0.0409
	(0.0488)	(0.164)	(0.280)	(0.0655)	(0.0578)	(0.0676)
Constant	3.278 ***	6.881 ***	−1.099	5.384 ***	6.736 ***	4.129 ***
	(0.181)	(1.000)	(0.997)	(0.722)	(0.745)	(0.232)
Individual characteristics	Yes	Yes	Yes	Yes	Yes	Yes
Household characteristics	Yes	Yes	Yes	Yes	Yes	Yes
Production characteristics	Yes	Yes	Yes	Yes	Yes	Yes
Observations	200	202	150	200	202	150
R-squared	0.352	0.504	0.056	0.107	0.087	0.036

Note: *** *p* < 0.01. Robust standard errors are given in parentheses.

**Table 5:** Mechanism testing.

	OLS			Logit		
Variables	(1)	(2)	(3)	(4)	(5)	(6)
	Inform	Market	Precise	Inform	Market	Precise
Digital	0.316 *	0.130 **	0.422 ***	0.744 ***	0.877 **	0.683 ***
	(0.164)	(0.0580)	(0.0468)	(0.286)	(0.405)	(0.1988)
Constant	3.386 ***	0.334	−0.657 ***	−0.295	−1.698	1.8.96
	(0.676)	(0.240)	(0.193)	(1.958)	(1.414)	(1.797)
Observations	552	552	552	552	552	552
R-squared	0.131	0.066	0.149	/	/	/

Note: * *p* < 0.1, ** *p* < 0.05, and *** *p* < 0.01. Robust standard errors are given in parentheses.

## Data Availability

The original contributions presented in this study are included in the article. Further inquiries can be directed to the corresponding author.
